# The AMP-Foot 3, new generation propulsive prosthetic feet with explosive motion characteristics: design and validation

**DOI:** 10.1186/s12938-016-0285-8

**Published:** 2016-12-19

**Authors:** Pierre Cherelle, Victor Grosu, Manuel Cestari, Bram Vanderborght, Dirk Lefeber

**Affiliations:** 1 Department of Mechanical Engineering, VUB, Pleinlaan 2, 1050 Brussels, Belgium; 2 Center of Automation and Robotics (UPM-CSIC), Arganda del Rey, 28500 Madrid, Spain

**Keywords:** Bionic feet, Prosthetics, Compliant actuation

## Abstract

The last decades, rehabilitation has become a challenging context for mechatronical engineering. From the state-of-the-art it is seen that the field of prosthetics offers very promising perspectives to roboticist. Today’s prosthetic feet tend to improve amputee walking experience by delivering the necessary push-off forces while walking. Therefore, several new types of (compliant) actuators are developed in order to fulfill the torque and power requirements of a sound ankle-foot complex with minimized power consumption. At the Vrije Universiteit Brussel, the Robotics and Multibody Mechanics research group puts a lot of effort in the design and development of new bionic feet. In 2013, the Ankle Mimicking Prosthetic (AMP-) Foot 2, as a proof-of-concept, showed the advantage of using the explosive elastic actuator capable of delivering the full ankle torques ($\pm 120$ Nm) and power ($\pm 250$ W) with only a 60 W motor. In this article, the authors present the AMP-Foot 3, using an improved actuation method and using two locking mechanisms for improved energy storage during walking. The article focusses on the mechanical design of the device and validation of its working principle.

## Background

The past decades, researchers have been studying pathological and non-pathological gait to understand the human ankle-foot function during walking. These efforts resulted in the development of new lower limb prosthetic devices aiming at raising the 3C-level (control, comfort and cosmetics) of amputees, each with slightly different characteristics. Thanks to the technological advances in computer aided design (CAD) and mechatronics, challenges in this field have become an important source of interest for roboticists. Today’s state-of-the-art in propulsive transtibial prostheses consist of no more than 23 devices that can be categorized based on their actuation principle as presented in [1]. From these, 16 prototypes have been developed in the USA [2–5], 5 in Belgium [6–8] and 1 in China [9]. Pioneers in the field are undoubtedly the research teams of Herr et al. (MIT—USA) [10–12], Sugar et al. (ASU—USA) [13–15] and Goldfarb et al. (Vanderbilt) [16, 17]. Yet 2 companies have emerged from these research centers, namely iWalk and SpringActive, bringing their know-how to the American market. Currently, most of the bionic feet are still on a research level, but show promising results and a preview of tomorrow’s commercial prosthetic devices.

At the Vrije Universiteit Brussels, a new type of actuation system has been developed for use in ankle-foot prostheses, named the explosive elastic actuator (EEA) [1]. The EEA consists of a spring behind a locking mechanism placed in series with a series elastic actuator (SEA). This catapult-like mechanism is based on the use of stored energy to hurl a payload, without the use of an explosive. The EEA therefore has the advantage of storing energy and release it when needed. This type of explosive motions are widely used in e.g. jumping [18], kicking [19], throwing [20] and hammering robots [21]. The torque requirements of the EEA are similar to the SEA in prosthetic feet. But by using a locking mechanism, the motor can provide its work during a longer period of time (typically 2–3 times for a prosthetic ankle), reducing by the same amount the actuator’s speed and power. This new type of actuation has proven its effectiveness with the Ankle Mimicking Prosthetic (AMP-) Foot 2 [6, 22, 23].

**Fig. 1:**
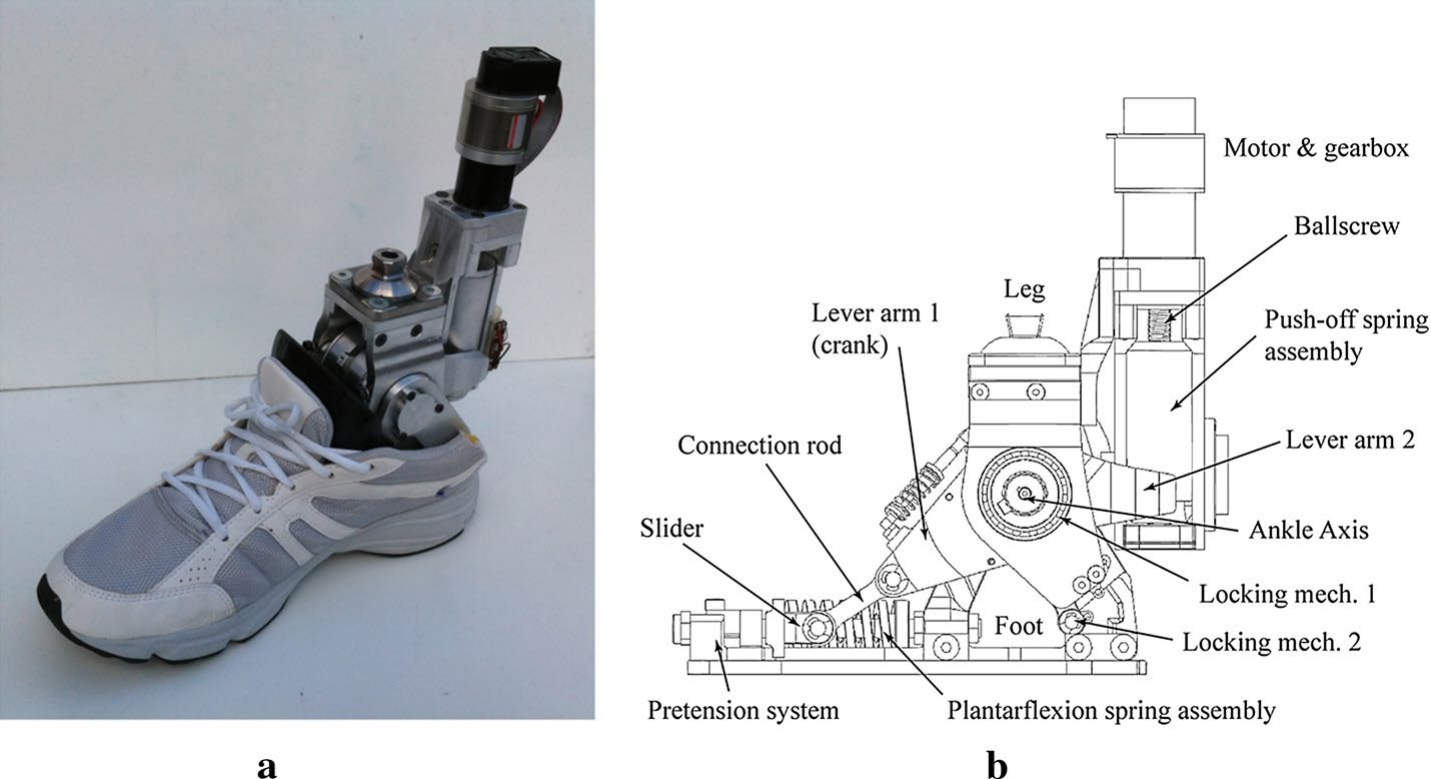
**a** Picture of the AMP-Foot 3. **b** The AMP-Foot 3 essential parts

In this article, the authors present their latest research prototype, the AMP-Foot 3 shown in Fig. 1. The prosthesis’ design is described and results of experiments with an amputee are presented. The novelty of this work relies in the use of 2 locking mechanism to improve the energy storage of the device compared to its predecessor, the AMP-Foot 2. Also, unlike in the previous prototype, no cables have been used. Instead a compliant crank-slider mechanism has been chosen to transmit the propulsion forces and torques to the ankle of the device. At first the concept behind the AMP-Foot 3, its working principle, mechanical design and electronics design are described in depth. Further the experimental validation of the prosthesis is presented by means of treadmill experiments with an amputee. Conclusions and future work will close the article.

## The AMP-Foot 3—development

In this section the development and working principle of the AMP-Foot 3 is presented.

### An energy efficient concept

The main objective of this research is the implemetation of the ‘principle of optimal power distribution’ [6] into a prosthetic foot, i.e. retrieve as much energy as possible from the gait and to incorporate an electric actuator with minimized power consumption. As shown in [6] and [23] the required output power can be decreased significantly by using the explosive elastic actuation princple. Unlike a regular SEA, the torque output can be provided during a longer lapse of time, therefore decreasing the electric drive’s speed, thus its power requirements.

Obviously, the AMP-Foot 3 predecessors are the AMP-Foot 2. But the new prototype is not just a redesign. The authors have improved its mechanics, functionality and decreased its power requirements by adding an extra, new locking mechanism to the system. For more information about the mechanical design and working principle of the AMP-Foot 2, the authors refer to [6] and [23].

### Working principle

**Fig. 2:**
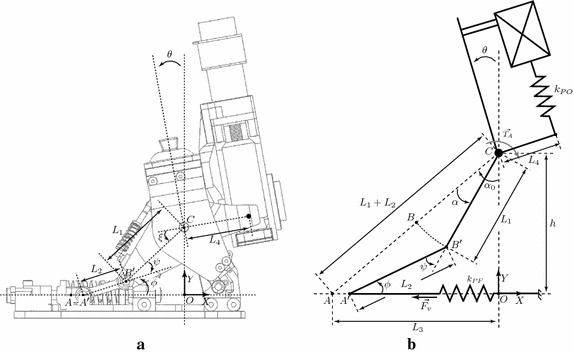
**a**, **b** The AMP-Foot 3 prototype schematics

In Figs. 1 and 2, the essential parts of the AMP-Foot 3 are represented. The device consists of four bodies pivoting around a common axis (the ankle axis-point *C*), i.e. the leg, the foot and two lever arms (depicted as lever arm 1 and 2). The motor, gearbox and ballscrew assembly are fixed to the leg. The system also comprises 2 springs sets: a plantarflexion (PF) and a push-off (PO) spring set. The PF spring set is placed between the foot and the slider of a crank-slider mechanism (point $A^{\prime}$) and is used to store and release motion energy. Lever arm 1 represents the crank of the latter while the connection rod is placed between the lever (point $B^{\prime}$) and the slider (point $A^{\prime}$). It is through this compliant crank-slider mechanism that forces from the leg and motor are transmitted to the foot. The reason for choosing a linkage mechanism compared to a cables and pulley system (as used in the AMP-Foot 2) is to improve the reliability of the system. The push-off spring on the other hand is placed in a tube between the motor-ballscrew assembly and a fixed point (*D*) on lever arm 2. The main idea behind the AMP-Foot 3 is to store motion energy in the PF springs, while a low power actuator compresses the PO springs without affecting the ankle joint. When push-off is needed, the energy stored in the PO spring is released and added to the energy stored in the PF springs assembly. This sudden addition of energy is hereby fed to the ankle joint and thus provides the propulsive forces and torques desired during walking. As mentioned, the AMP-Foot 3 makes use of 2 locking mechanisms. Locking mechanism 1 is a resettable overrunning system providing a one way clutch connection between the two lever arms. This locking mechanism is used to maximize the stored motion energy during midstance compared with the AMP-Foot 2. A second advantage of this locking mechanism is a better mimicking of the human gait characteristics by allowing a change in PF spring rest position after the foot is stabilized and the ankle enters its dorsiflexion phase. Locking mechanism 2 provides a rigid connection between the leg and the lever arm when energy is injected into the system by the electric drive. Comparable to the one used in the AMP-Foot 2, its role is disengaging the electric actuator from the ankle joint when loading the PO spring. More information on the locking mechanisms’ working principles is given further in the text. To maintain a consistent notation through the article, symbols and names used in Figs. 1 and 2 are described as:

1 $$\begin{aligned} L_1&= \text {distance between ankle axis (C)} \,\, \text {and point }B^{\prime}. \nonumber \\ L_2& = \text {distance between point }A^{\prime} \text{and point }B^{\prime}. \nonumber \\ L_3& = \sqrt{(L_1+L_2)^2-h^2} \end{aligned}$$

2 $$\begin{aligned} h&= \text {distance between ankle axis (C)} \,\, \text {and the origin O.}\nonumber \\ L_4&= \text {distance between} \,\,\text {ankle axis (C) and point D.}\nonumber \\ \theta &= \text {angle between foot and leg.}\nonumber \\ \xi &= \text {angle between lever arm 1 and 2}\nonumber \\ \alpha _0 = \text {angle between lever arm 1 and foot when} \,\, \text {the crank-slider is not loaded.}\nonumber \\ \alpha &= \theta + \xi = \text {angle rotation of lever arm 1.} \end{aligned}$$

$$\begin{aligned} \psi &= \text {angle between lever arm 1} \,\, \text {and connection rod.}\\ \phi &= \text {angle between connection rod and slider.}\\ k_{PF} &= \text {plantarflexion spring assembly stiffness.} \\ k_{PO} &= \text {push-off spring stiffness.} \\ \vec {F_v}&= \text {force exerted by the plantarflexion spring.}\\ \vec {T_A}&= \text {torque applied to the ankle joint.} \end{aligned}$$

**Fig. 3:**
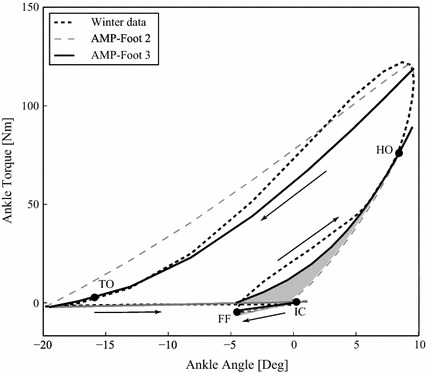
The AMP-Foot 3 prototype simulated angle-torque characteristic compared to the reference data [24] and the AMP-Foot 2 simulated data. The* shaded area* represents the extra energy that can be stored thanks to the use of locking mechanism 1 compared to the AMP-Foot 2 prototype. This area represents approximately $5\,J$

A detailed description of the behavior of the AMP-Foot 3 using the principle of optimal power distribution is given by illustrating one complete gait cycle. To do this, on gait cycle is divided into its 5 main phases (shown in Fig. 3):

$$\begin{aligned} \begin{array}{ll} Phase 1 : &{} From\;initial\;contact\;(IC) to {}foot\;flat (FF).\\ Phase 2 : &{} From\;FF\;to\;heel\;off\;(HO).\\ Phase 3 : &{} At\;heel\;off\;(HO).\\ Phase 4 : &{} From\;HO\;to\;toe\;off\;(TO).\\ Phase 5 : &{} Swing\;phase.\\ \end{array} \end{aligned}$$

The gait cycle starts with a *controlled plantarflexion* from initial contact (IC) to foot flat (FF) produced by muscles as the *Tibialis Anterior*. This is followed by a *controlled dorsiflexion* phase ending in *push-off* at heel off (HO) during which propulsive forces are generated by the calf muscles. During *late stance*, the torque produced by the ankle decreases until the leg enters the *swing* phase at toe off (TO). Once the leg is engaged in the swing phase, the foot resets to prepare for a new step. The working principle of the prosthetic device during each phase is explained here under.

#### From IC to FF

A step is initiated by touching the ground with the heel. During this phase the foot rotates with respect to the leg, until $\theta$ reaches approximately $-5$°. During this phase lever arm 2 is fixed to the leg. The resettable one way clutch placed between lever 1 (noted as $L_1$) and 2 (depicted as $L_4$) allows the leg to move backwards (until maximum $12$°) without moving lever arm 1. Therefore $\xi$, being the angle between lever arm 1 and 2, increases. The small negative required torque in this phase is provided by two small tension springs attached between the leg and the foot (not shown in the figure). Because the range of motion is small (a few degrees) and the pretension of these small tension springs is high, their torque characteristic is highly linear and therefore can be modeled as a torsional spring with stiffness $k_T = \pm 50$ Nm/rad. The torque is then calculated as:

3 $$\begin{aligned} T_A = k_T \theta \end{aligned}$$

During this period the electrical drive starts loading the PO spring. Since the motor is attached to the leg and lever arm is locked to the leg, the PO spring is loaded without delivering torque to the ankle joint. Therefore the prosthesis is not affected by the forces generated by the actuator.

#### From (FF) to heel off (HO)

When the foot stabilizes at FF, the leg moves from approximately $\theta = -5$° to +10°. Once the leg starts moving in this direction, the resettable overrunning mechanism is engaged instantaneously, fixing hereby lever arm 1 to lever arm 2 (which itself is fixed to the leg because of the second locking mechanism). Because of this, the two tension springs elongated previously in *phase 1* are fixed and therefore do not provide any torque to the ankle joint anymore. One can say that their action is removed from the system (while they are still elongated). These springs will remain in this state until the overrunning mechanism is disengaged at the beginning of the swing phase. The energy stored in these springs will then serve for resetting the ankle-foot prosthesis. The lever follows the movement of the leg and torque is generated at the anke joint by actioning the compliant crank slider mechanism. Moving the leg forward elongates the plantarflexion (PF) springs. Thanks to the use of locking mechanism 1, motion energy is stored in the PF springs as soon as the ankle goes in dorsiflexion at approximately $-5$° (depending on the walking pattern of the user). This corresponds on average to an additional energy storage between 5 and 10 J compared to the AMP-Foot 2 in which the motion energy of the mid-stance phase could only be stored from 0°. During this phase, based on Fig. 2 the torque at the ankle is given by Eq. (4).

4 $$\begin{aligned} T_A = L_1 |\vec {AA{^{\prime}}}| k_{pf} \cos {\phi } \sin {\psi } \end{aligned}$$

in which:

5 $$\begin{aligned} \sin {\psi }&= \sqrt{1-\left(\frac{\vec {CB^{\prime}} . \vec \, {B^{\prime}A^{\prime}}}{L_1L_2}\right)^2 } \end{aligned}$$

6 $$\begin{aligned} \vec {OA}&= (-L_3 , 0) \end{aligned}$$

7 $$\begin{aligned} \vec {OB}&= (-L_1 \sin {\alpha _0} ,\quad h - L_1 \cos {\alpha _0})\end{aligned}$$

8 $$\begin{aligned} \vec {OC}&= (0 , h)\end{aligned}$$

9 $$\begin{aligned} \vec {OA^{\prime}}&= (-L_1 \sin {(\alpha _0-\alpha )} - \sqrt{ L_2^2 - (h - L_1 \cos {(\alpha _0-\alpha )})^2} , 0) \end{aligned}$$

10 $$\begin{aligned} \vec {OB^{\prime}}& = (-L_1 \sin {(\alpha _0-\alpha )} ,\,\, h - L_1 \cos {(\alpha _0-\alpha )})\end{aligned}$$

11 $$\begin{aligned} |\vec {AA^{\prime}}|&= L_3-L_1 \sin {(\alpha _0-\alpha )} - L_2 \cos {\phi } \end{aligned}$$

During this phase the motor is still injecting energy into the system by loading the PO spring without affecting the behavior of the device.

#### At heel off (HO)

Because the angle between the PO spring and the lever arm is fixed at $90$°, the torque exerted by the PO spring (no pretension) on the lever arm is given by

12 $$\begin{aligned} T_{EEA} = k_{PO}l_2L_4 \end{aligned}$$

with $T_{EEA}$ representing the torque applied to lever arm 2 by the EEA and $l_2$ the compression of the PO spring.

The torque $T_A$ provided by the plantar flexion spring on lever arm 1 is given by Eq. (4). At HO, locking mechanism 2 is forced to unlocked and all the energy stored into the PO spring is fed to the system. Since $T_A \le T_{EEA}$, both PF and HO springs tend to rotate the lever arm with an angle $\chi$ to a new equilibrium position. $T_A$ and $T_{EEA}$ respectively evolve to new values $T^{\prime}_A$ and $T^{\prime}_{EEA}$ such that $T^{\prime}_A = T^{\prime}_{EEA} = T'$ with $T^{\prime} \ge T_A$ and $T^{\prime} \le T_{EEA}$. The torque at the ankle is then calculated with Eq. (4) taking into account the extra angle $\chi$. In other words $(\alpha _0-\alpha )$ becomes $(\alpha _0-\alpha - \chi )$.

The effect of this is a virtually instantaneous increase in torque and decrease in stiffness of the ankle joint. This is shown in Fig. 3 which represents the torque-angle characteristic of an intact ankle according to gait analysis conducted by Winter [24] and of the simulated AMP-Foot 3 behavior.

#### From HO to toe off (TO)

In the last phase of stance, the torque is decreasing until toe off (TO) occurs at $\theta = -20$°. Since the plantarflexion and push-off springs are now connected in series, the rest position of the system has changed according to the elongation and restlength of the PO spring. As a result of this a new equilibrium position is set to approximately $\theta = -20$°. The actuator is still working during this phase.

#### Swing phase

After TO, the leg enters into the so called swing phase in which the whole system is reset, including locking mechanism 1. How this is achieved will be explaind further in the text. While the motor turns in the opposite direction to bring the ballscrew mechanism back to its initial position, the 2 tension springs used in *phase 1* are reactivated and its stored energy is used to set $\theta$ back to 0° and to close the four bar linkage locking mechanism (locking mechanism 2). At this moment, the device is ready to undertake a new step.

### Mechanical design

In this section a detailed description of the mechancal design of the AMP-Foot 3 is given. At first the design criteria and general parameters are given. Then the EEA is presented followed by an explenation of the two locking mechanism designs.

#### Design criteria and general parameters

**Table 1:** Lever arm and springs

$L_1$ = 70 mm	$L_2$ = 40 mm
$L_3$ = 103 mm	$L_4$ = 60 mm
$k_{PF}$ = 300 N/mm	$k_{PO}$ = 180 N/mm

**Fig. 4:**
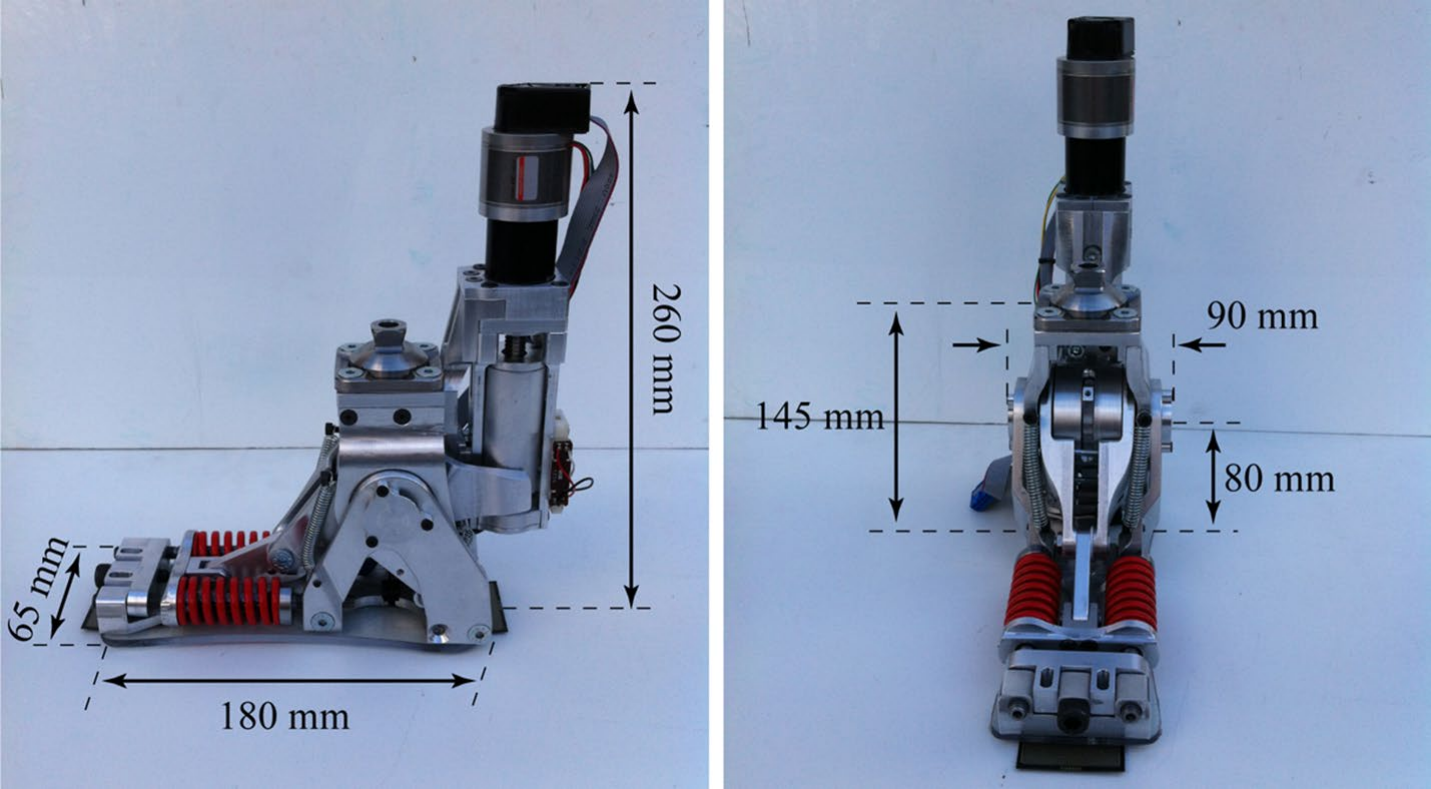
The AMP-Foot 3 prototype

A 75 kg subject walking at normal cadence on ground level produces a maximum joint torque at the ankle of appromately 120 Nm [24]. This has been taken as a criterion. Moreover, an ankle articulation has a moving range from approximately $+ 10$° at maximal dorsiflexion to $- 20$° at maximal plantarflexion. Therefore a moving range of $-30$° to $+20$° has been chosen for the system to fulfil the requirements of the ankle anatomy. The foot is made to match a European size between 41 and 45 with a ankle height of approximately 80 mm. In Fig. 4 the dimensions of the AMP-Foot 3 are depicted. With this design, the prosthesis fits in a shoe which is significantly more comfortable for the amputee. The connection with the socket of the subject is provided with an Otto-Bock pyramid adaptor. The device has a weight of approximately 3 kg (not including batteries which are currently worn at the hip), which is still acceptable according to the person subjected to the clinical trials. The length of the lever arms and springs stiffnesses used in Fig. 2 are given in Table 1.

#### The explosive elastic actuator (EEA)

**Table 2:** Motor and transmissions

Motor	Maxon ECi 40–50 W
	$T_{cont.}$ = 46.6 mNm
	$T_{peak}$ = 100 mNm
Transmission	Maxon GP32BZ
Stage 1	i = 5.8:1
Transmission	Maxon ballscrew GP32S
Stage 2	$\phi$ 10 x 2 mm
	$$\eta _{transmission1 \& 2} = \pm75 \%$$

To fulfil the requirements of a sound ankle, a motor with a high ‘power and strength to weight’ ratio and mechanical efficiency is needed. Based on peak torque and power estimation, a Maxon ECi-40 motor (50 W) was chosen with its corresponding gearbox and ballscrew assembly, as described in Table 2. The placement of the motor and its necessary electronics have been chosen to optimise compactness of the system.

#### Locking mechanisms

As mentioned before, the system comprises two locking mechanisms: a resettable one-way clutch and a four bar linkage locking mechanism. Both of them are critical to the well functioning of the device.

- *Locking mechanism 1* The novelty of the AMP-Foot 3 prototype relies in the design and use of this locking mechanism. To enable a change in rest position of the plantarflexion spring during the first phase of gait (from IC to FF) a resettable continuous one way clutch has been developed to decouple the two lever arms. The locking mechanism is based on the well known freewheel principle consisting of spring-loaded steel rollers inside a hardened cylinder. Rotating in one direction, the rollers lock with the outer race making it rotate in unison. If rotating in the other direction, the steel rollers will slip inside the cylinder without transmitting torque. In addition a lever is placed next to the clutch offering the possibility to push the rollers against the springs, disengaging the clutch and allowing it to rotate freely in both directions. However it should be noted that an energy efficient disengagement is only possible when the rollers are not wedged in the cylinder. As such, the presented resettable clutch mechanism is a rotative, continuous, one way locking without backlash with the possibility to be disengaged (and reset) when unloaded (at the very beginning of the swing phase). These features fits completely the requirements of the AMP-Foot 3 prototype. To ensure proper unlocking, a servomotor in series with a compression spring is attached to the reset lever of the clutch. During the gait (when the locking mechanism is loaded) the spring is compressed until the servomotor reaches a singular position. The principle is actually a small scale EEA. Once the load is removed from the clutch, and because the spring is compressed, the locking is disengaged instantaneously. This overrunning clutch is designed to keep up to 160 Nm of torque. Advantages of using this mechanism is the fact more energy can be stored in the PF spring assembly during mid-stance and its potential to adapt naturally to different walking speeds and slopes. Disadvantages are the extra weight and volume.
- *Locking mechanism 2* The second locking mechanism uses the same principle as the one used in the AMP-Foot 2. This mechanism is placed between the leg and the second lever arm in order to decouple the series elastic actuator (SEA) from the ankle joint. Because of this, it must be able to withstand high forces while being as compact and lightweight as possible. The crucial and challenging part is that the mechanism must be unlocked when bearing its maximum load and last but not least, this unlocking must require a minimum of energy. Fortunately, the lever arm has to be locked to the leg at a fixed angle. With these requirements it has been chosen to work with a four bar linkage moving in and out of a singular position. This principle has already proved its effectiveness in [6]. However, unlike in the AMP-Foot 2, the unlocking of the four bar linkage is not triggered by a servo motor. This time unlocking happens by moving the leg forward against a mechanical stop. This mechanical stop can be positioned as such that the unlocking angle can be adapted. This way, the authors have shown that unlocking, even under maximum load, can be done from the motion of the user.

The AMP-Foot 3 is, at the foot, equipped with a custom made loadcell which allows a force measurement with a resolution of $\pm 5$ N and the elongation of the PO springs is measured with a linear potentiometer. To measure the position of the lever arm, and the leg with respect to the foot, two absolute magnetic encoders (Austria Micro Systems AS5055) are used with a resolution of $\pm 0.08$°. While the magnets of the encoders are glued to the ankle axis (which is fixed to lever arm 2) and the leg, the two hall sensors are fixed on the foot. As a result of this, the resulting torque at the ankle can be calculated using the mathematical model of the mechanical system which has been discussed before. To detect the important triggers during the stance phase (IC, FF, HO, TO), two force sensing resistors (FSR) are placed on the foot sole: one at the heel and one at the toes. These triggers will be used to control the motor and to unlock locking mechanism 1. A current sensor is also used to measure the current sent to the motor. This information serves essentially in the low level control of the device. In addition a 6 DOF IMU has been incorporated in the foot for future control perspectives.

**Fig. 5:**
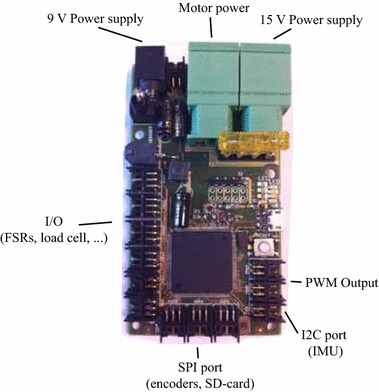
Custom made microcontroller board

The electronics of the prosthesis consist of a Maxon Escon controller, that handles the low level control of the motor, and a custom made microcontroller board (shown in Fig. 5) based on the Atmel SAM3X8E ARM Cortex-M3 CPU managing the high level control and gait detection. All the data from the sensory network are recorded on an SD card.

The ‘Optimal Power Distribution’ has already shown its impact on the simplicity of the control of the prosthesis with the AMP-Foot 2 prototype [6, 23]. Since the output axis of the actuator is not directly controlling the ankle axis, a very simple control strategy can be used. Currently, the maxon ESCON controller only uses a PID current loop. In addition, the high level control detects walking patterns of the subject and, in function of this, sends the appropriate information to the ESCON controller. For the conducted experiments in this article, the current value sent to the motor controller is fixed and corresponds to the approximate requirements of walking on level ground at the subject’s self selected speed. Future control perspectives are mentioned in the concluding section.

**Table 3:** Battery specifications

Type	LiPo
Nominal voltage	14.8 V (4 cells)
Capacity	5000 mAh
Discharge capacity	250 A (continuous)
	500 A (burst)
Size	155 x 47 x 29 mm
Mass	556 g

As power source, an oversized battery has been used to avoid any risk of power failure during the experiments. The battery specifications are listed in Table 3. This has been used during the experiments.

## The AMP-Foot 3—validation

In this section, the authors present the captured data of an amputee walking with the AMP-Foot 3 prosthesis.

### Methods

The AMP-Foot 3 prototype was tested with Mr. A. The subject being a transfemoral amputee, he has been using his own knee prosthesis (Össur Mauch Knee) together with the AMP-Foot 3. For the validation of the device, 3 experiments have been conducted. The first two experiments, Mr. A. was asked to walk on a treadmill at self selected speed with his own prosthesis (Össur Modular III) and with the AMP-Foot 3 in passive mode (without actuation). The third conducted experiment was identical but this time with actuation of the prosthesis, and thus push-off generation.

### Results and discussion

During the first experiment, Mr. A was asked to walk at self selected speed with his own prosthesis in order to compare with his self selected speed wearing the AMP-Foot 3. The subject appeared to feel most comfortable at a speed of about 3.5 km/h. Then the same experiment was repeated with the AMP-Foot 3 in its passive mode (meaning the electric motor was not used) and showed an improvement of 0.5 km/h resulting in a self selected speed of 4.0 km/h. According to Mr. A., he felt more comfortable while walking thanks to the change in rest position of the PF spring in the first phases of gait (after FF—due to locking mechanism 1) and the fact the AMP-Foot 3 is an efficient energy storing and returning (ESR) foot when used in passive mode compared to his own Modular III prosthesis. Indeed, this locking mechanism presents interesting assets such as passive self adaptation to different walking speeds and slopes which our subject noticed rapidely. However, this article only focusses on the validation of the AMP-Foot 3 concept prototype. The fact the AMP-Foot 3 can be used in passive mode remains a very interesting asset in case the battery would be discharged. In such situation the prosthesis can still be used in a safe way but without producing extra propulsive forces to the wearer.

**Fig. 6:**
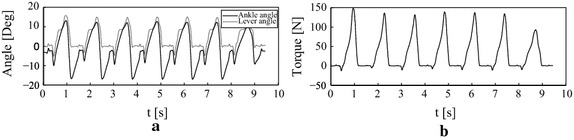
Time-based data of level ground walking at 4.7  km/h with the AMP-Foot 3. **a** Ankle and lever angle vs. time. **b** Ankle torque vs. time

**Fig. 7:**
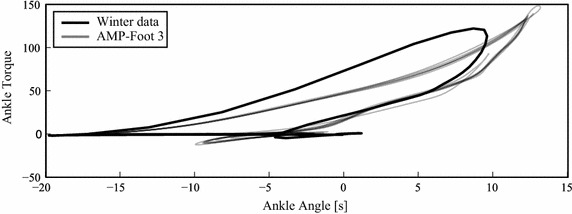
Torque-angle characteristic of the AMP-Foot 3 while walking at self selected speed (4.7 km/h)

Again the same experiment was repeated, but this time with actuation which revealed a comfortable self selected speed of 4.7 km/h. In Fig. 6, the time-based data of level ground walking at 4.7 km/h is shown. Fig. 6a represents the ankle and lever ankle of the AMP-Foot 3 and Fig. 6b is the deployed ankle torque while walking. Mr. A had a step length of approximately 1.5 m while walking on a treadmill. It can be noticed that the subject has a wide plantarflexion angle (on average $-10$°) during the ‘HS to FF’ phase compared to the reference data [24] (approximately $-5$°). This explains why Mr. A. particularly appreciates the change in rest position of the PF spring in this first phase by the action of locking mechanism 1. One can also notice that while loading the PF spring in midstance, the lever arm and ankle angle differs slightly. This is due to play in the four bar linkage which locks both moving parts. However it can be seen that the lever arm angle is slightly bigger than the ankle angle which means that the PO spring assembly produces more torque on the lever than the PF spring. This is a necessary condition to provide push-off to the amputee. At the end of mid stance, the energy stored in the PO spring is released by releasing the four bar locking mechanism. Therefore the lever finds a new equilibrium position. In Fig. 7 the corresponding torque characteristic of the AMP-Foot is shown. During the experiments it was noted that during some steps no extra power was provided. This is due to the fact the four bar locking mechanism did not unlock itself. Unlike in the AMP-Foot 2, the unlocking is done in a passive way in the AMP-Foot 3. However after using the prosthesis for approximately 30 min, Mr. A. did better understand its way of working and started to adapt himself for the proper use of the AMP-Foot prototype. As a matter of fact, changing from a passive, non-articulated carbon prosthesis to an articulated, powered system needs some serious adaptation of the wearer.

**Fig. 8:**
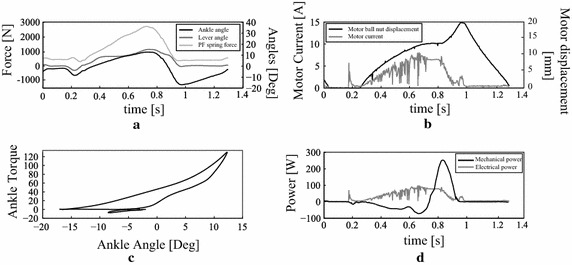
Time-based data of level ground walking at self selected speed (4.7 km/h) with the AMP-Foot 3 during one step. **a** Ankle, lever angle and PF spring force vs. time. **b** Motor ball nut displacement and current consumption vs. time. **c** Ankle torque vs. ankle angle. **d** Electrical and mechanical power vs. time

In Fig. 8a one-step representative is shown of level ground walking at self selected speed (4.7 km/h) with the AMP-Foot 3. Figure 8a represents the Ankle angle, lever arm angle and the PF force during one stride. Because of the mechanical design of locking mechanism 1 (acting between the two lever arms), it is seen that the lever doesn’t follow the ankle angle at the very beginning of the gait cycle. This explains the difference between the lever angle and ankle angle during the dorsiflexion phase. They follow each other until the PO springs get tensioned and released. At push-off the two angles show major differences until the system is reset during the swing phase, bringing both the foot and the lever to approximately the same angle value. In Fig. 8b the motor displacement and current consumption is shown. It can be seen that the motor compresses the PO spring until approximately 11 mm while the motor consumes up to approximately 6 A. When the four bar linkage is unlocked, the motor’s ballnut moves rapidely to 15 mm while the motor current decreases. Figure 8c shows the torque characteristic of the corresponding step. As noticed before, it can be seen that Mr. A. has a wide plantarflexion angle before FF occurs. Furthermore it is clear that the torque-angle characteristic represents a loop to be followed anticlockwise, which indicates energy production. The maximum plantarflexion angle at the end of stance goes to approximately $-17$° before the toes are lifted from the ground and the AMP-Foot enters the swing phase. During swing, the complete system undergoes a hardware reset to prepare for the next step. To close the validation of the AMP-Foot 3, the electrical and mechanical power of the device is shown in Fig. 8d. From the mechanical point of view it is clear that the AMP-Foot 3 respects the needs of an amputee when considering Winter’s gait analysis as reference data [24]. From the electrical point of view it can be seen that the electric power increases while compressing the PO spring. At maximum compression a peak power of slightly less then 100 W is provided. It should be noted however that the RMS power is about 55.5 W. As explained before, the main idea is to provide the power during the complete stance phase, which is not exactly followed here. The reason for this is because of limitations imposed by the manufacturer of the Maxon ESCON controllers. Better tuning of these low level controllers may improve the power consumption of the device. During the one-step example shown in Fig. 8, integration of the mechanical power curve shows that approximately 13 J was stored in the PF spring assembly during early stance and that about 26 J of energy is delivered at push-off which corresponds to the requirements of a sound ankle.

## Conclusions and future work

**Fig. 9:**
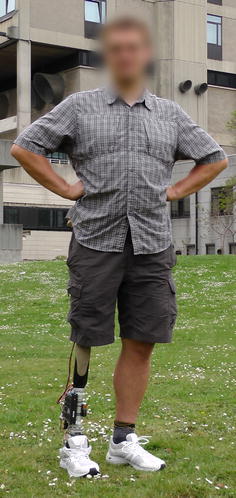
Picture of Mr. A. wearing the AMP-Foot 3

**Fig. 10:**
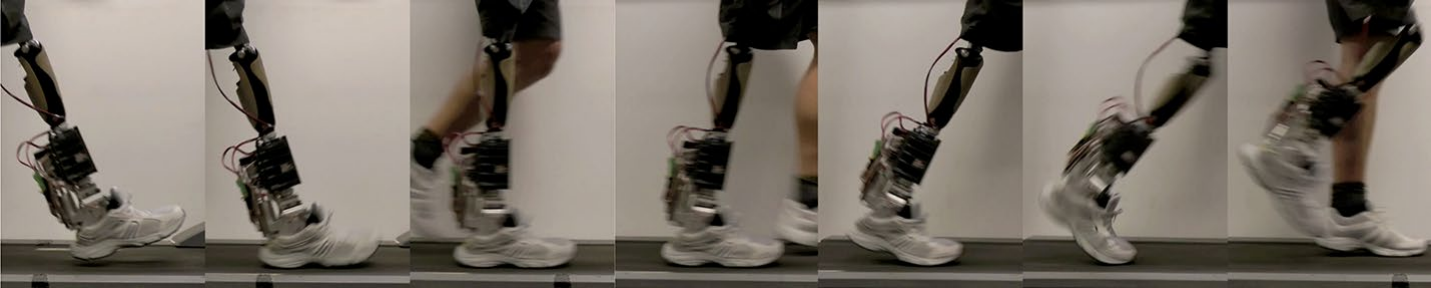
Walking sequence with the AMP-Foot 3

In this article, the authors have proposed a new design of an energy efficient powered transtibial prosthesis mimicking able-bodied ankle behavior, the AMP-Foot 3, combining the explosive elastic actuation and an extra locking mechanism. The innovation of this study is to gather energy from motion during the controlled dorsiflexion with a PF spring while storing energy produced by a low power electric motor into a PO spring. This energy is then released at a favorable time for push-off thanks to the use of a locking system. The AMP-Foot 3 mechanical design is presented and the prototype is validated by means of experiments with an amputee (Figs. 9, 10). It can be concluded that the AMP-Foot 3 is capable of providing a 75 kg amputee with the propulsive forces and torques of a sound ankle thanks to the use of the EEA. Although its mechanical properties showed positive results, its control (low and high level) needs to be improved to decrease the overall power consumption and to accommodate for different functions. However it is noted that the average power produced by the AMP-Foot 3 is only 55.5 W. A drawback of the system is its weight of approximately 3 kg, which is still acceptable for a prosthetic foot. Future work will consist of improving its low level control, adding a multi-functional high level control and gait detection system. The potential benefits of using the extra locking system to provide automatic adaptation to different walking speeds and slopes will also be further analyzed.

## Abreviations

AMP-Foot: Ankle Mimicking Prosthetic Foot
3C: control, comfort and cosmetics
CAD: computer aided design
EEA: explosive elastic actuator
SEA: series elastic actuator
PF: plantarflexion
PO: push-off
IC: initial contact
FF: foot flat
HO: heel off
TO: toe off
ESR: energy storing and returning
RMS: root mean square
